# Microglial Dysfunction in Neurodegenerative Diseases via RIPK1 and ROS

**DOI:** 10.3390/antiox11112201

**Published:** 2022-11-07

**Authors:** Qiaoyan Wu, Chengyu Zou

**Affiliations:** 1 Interdisciplinary Research Center on Biology and Chemistry, Shanghai Institute of Organic Chemistry, Chinese Academy of Sciences, 100 Haike Rd, Pudong District, Shanghai 201210, China; 2 Shanghai Key Laboratory of Aging Studies, 100 Haike Rd, Pudong District, Shanghai 201210, China

**Keywords:** microglia, neurodegenerative diseases, NF-κB activation, RIPK1, ROS

## Abstract

Microglial dysfunction is a major contributor to the pathogenesis of multiple neurodegenerative diseases. The neurotoxicity of microglia associated with oxidative stress largely depends on NF-κB pathway activation, which promotes the production and release of microglial proinflammatory cytokines and chemokines. In this review, we discuss the current literature on the essential role of the NF-κB pathway on microglial activation that exacerbates neurodegeneration, with a particular focus on RIPK1 kinase activity-dependent microglial dysfunction. As upregulated RIPK1 kinase activity is associated with reactive oxygen species (ROS) accumulation in neurodegenerative diseases, we also discuss the current knowledge about the mechanistic links between RIPK1 activation and ROS generation. Given RIPK1 kinase activity and oxidative stress are closely regulated with each other in a vicious cycle, future studies are required to be conducted to fully understand how RIPK1 and ROS collude together to disturb microglial homeostasis that drives neurodegenerative pathogenesis.

## 1. Introduction

Microglia are the most common resident innate immune cells in the central nervous system (CNS), which have pivotal roles in sensing changes in the milieu of the brain, defending against infectious pathogens and noxious agents, and providing neuroprotection in neurodegeneration and maintaining brain homeostasis [1]. When a microbial invasion or injurious process happens in the brain, resting microglia are rapidly activated and undergo morphological changes in response to such stimulus [2]. Activated microglia can attenuate neuronal insults and repair damaged tissue by phagocytosing injurious agents or cell debris while releasing kings of inflammatory regulators to modulate the immune response [3]. However, microglia would be continuously activated if the inflammatory responses are not properly resolved. Hyperactivation of microglia leads to upregulated proinflammatory cytokines and large amounts of reactive oxygen species (ROS), which will accelerate neuronal injury and exacerbate pathological processes [4]. Consequently, the chronic proinflammatory microglia have changed from a protective phenotype to a detrimental one, resulting in progressive neurotoxic consequences [5].

Microglial dysfunction is one of the hallmarks and leading causes of neurodegenerative diseases [6]. Accumulating evidence indicates that microglia-regulated neuroinflammation and excessive ROS production are involved in the pathogenesis of many neurodegenerative diseases [7]. The most common neurodegenerative diseases, such as Alzheimer’s disease (AD), Parkinson’s disease (PD), Huntington’s disease (HD), and amyotrophic lateral sclerosis (ALS), are all characterized by aberrant aggregation of disease-causing proteins in the brain, which can directly activate microglia, trigger microglia-mediated neuroinflammation, and increase oxidative stress.

Amyloid-β (Aβ) deposits in AD can activate microglia through the interaction with several toll-like receptors (TLRs), including TLR2 and TLR4 [8], and triggering receptor expressed on myeloid cells 2 (TREM2) on the surface of microglia [9]. Likewise, the interaction between aggregated tau protein, another pathological hallmark of AD and tauopathies, and polyglutamine binding protein 1 (PQBP1), a polyglutamine tract amino acid sequence binding protein that is expressed in microglia, has been detected in vivo and in vitro [10]. Aβ and tau fibril-stimulated microglia exhibit upregulated NF-κB signaling, which is the key regulator of many canonical inflammatory pathways [11,12]. The NF-κB activation in microglia could trigger the release of a large variety of proinflammatory mediators, including interleukin-6 (IL-6), interleukin-1 beta (IL-1β), tumor necrosis factor-alpha (TNF-α), and interferon-gamma (IFN-γ) [13]. These inflammatory cytokines further activate other resting microglia and lead to acute inflammatory reactions with excessive ROS production [14]. In addition, TNF-α can also reactivate microglia in an autocrine mechanism through the NF-κB pathway [15], thus significantly inducing the up-regulation of Nos2 and proinflammatory gene expressions. Consequently, overactivated microglia secrete nitric oxide and proinflammatory cytokines, which are highly neurotoxic and dramatically reduce neuron viability and integrity by inducing large amounts of ROS [16].

In addition to the release of proinflammatory cytokines, activated microglia can produce chemokines to recruit monocytes and T lymphocytes across the blood–brain barrier. These peripheral immune cells can also play detrimental roles in CNS inflammation, which further contributes to the immune pathology of neurodegenerative diseases [17]. RNA-seq analysis has found that α-SYN stimulated microglia robustly upregulate the expression of plenty of chemokines and their receptors, including Ccl2, Ccl3, Ccl4, Ccl7, Ccl12, and Cxcl10, which are known to attract T cells and peripheral monocytes, among which CD8^+^ T cells and CD4^+^ T cells account for the majority of them [18]. CD8^+^ T cells mainly express the Ifng, granzyme (including Gzmb and Gzmk), and cathepsin W genes, while CD4^+^ T cells express high levels of Il2ra, Tnfrsf4, Ccr7, Bhlhe40, and Ifng. High expression of Ifng and Gzmb leads to potential cytotoxic activity. Microglia-mediated infiltration of peripheral immune cells and uncontrolled T cell activity in the CNS not only modulate chronic neuroinflammation but also result in severe tissue damage.

## 2. NF-κB Pathway Is Essential for Microglial Activation and Subsequent Neurotoxicity

Known to modulate the expression of many genes associated with glial activation, neuroinflammation, oxidative stress, neuronal development, and cell apoptosis in CNS [19], dysregulation of the nuclear factor-κB (NF-κB) signaling has been implicated in various neurodegenerative diseases. The NF-κB proteins, including NF-κB1 p50, NF-κB2 p52, RELA (also termed p65), RELB, and c-REL, are important nuclear transcription factors that express ubiquitously in a variety of CNS cell types, including microglia.

Numerous studies have found that proinflammatory microglia-mediated neurotoxicity in neurodegenerative diseases largely depends on the activation of the NF-κB pathway. Inhibition of NF-κB signaling selectively in microglia by chemicals can alleviate the production of pro-inflammation cytokines, chemokines, ROS, and nitric oxide, suppress LPS and other inflammatory trigger-induced microglial activation and attenuate microglia-mediated neuronal damage [20,21,22]. In a mouse model of ALS, NF-κB DNA-binding activity is predominately increased in microglia with disease progression. Inhibiting the NF-κB signaling through mutation of IκB that resists phosphorylation-induced proteasomal degradation in microglia rescues neuronal survival in vitro and in vivo, suggesting that the activation of classical NF-κB pathway, which drives the upregulated gene expression of proinflammatory cytokines, chemokines, adhesion molecules, and enzymes, regulates microglia to be converted into a proinflammatory and neurotoxic state [23]. Additionally, in pathological tau-treated microglia, NF-κB transactivation and the expression of many NF-κB target genes are upregulated. The activation of the microglial NF-κB pathway is also associated with enhanced cell movement, proliferation, and phagocytosis. Inhibition of NF-κB signaling through knocking out IKKβ slows down tau processing in microglia. It thus leads to reduced release of pathological tau species, decreased tau-induced neuronal toxicity, and potentially contributes to ameliorated cognitive deficits. These data together show that the canonical NF-κB pathway plays a critical role in microglia-mediated tau seeding and spreading [13].

CSB6B, as a CNS-safe pigment originally used in biochemical staining, has now been shown to significantly inhibit the expression of the main activator within NF-κB called p65 in microglia, subsequently preventing the expression of NLRP3 and decreasing cleaved caspase-1 and IL-1β [24]. In addition, reducing the binding activity between NF-κB and DNA by knocking down TRIM21 eliminates the increased expression of NLRP3 and cleaved caspase-1 in LPS-treated microglia and reduces the secretion of pro-inflammation cytokines such as TNF-α, IL-6, and IL-1β [25]. However, in neutrophils, the inhibition of NF-κB by pharmacologic or genetic means exacerbates NLRP3-dependent inflammation, as the IL-1β release of neutrophil is serine protease, other than caspase 1, dependent. NF-κB gene products inhibit serine protease activity [26]. Due to these opposite effects of NF-κB activation on microglia and neutrophil inflammatory responses, caution should be exerted on when to use long-term NF-κB inhibitors [27,28].

Microglia inflammatory progression is also mediated via the non-canonical NF-κB pathway. In experimental autoimmune encephalomyelitis (EAE), a mouse model of multiple sclerosis (MS), a much higher level of p52 in microglia is detected in the later stages of the pathological process. Conditionally deleting the NIK-encoding gene that blocks non-canonical NF-κB pathway activation in microglia inhibits microglial activation, which results in attenuated expression of chemokines, such as Cxcl9, Cxcl10, Cxcl11, and proinflammatory cytokines. It therefore decreases the number of CNS-infiltrating T cells and ameliorates EAE disease scores in the late phase of MS. These results indicate that the non-canonical NF-κB pathway is activated and essential for microglia to promote neuroinflammation during MS pathogenesis [29].

## 3. RIPK1 Mediates NF-κB Pathway Activation

Given the accumulating evidence that NF-κB pathway activation plays a critical role in the microglia-mediated inflammation that increases oxidative stress and promotes neuronal dysfunctions it is, therefore, imperative to illustrate potential molecular targets against NF-κB pathway activation for the treatments of neurodegenerative diseases. Receptor-interacting protein kinase 1 (RIPK1) is a serine-threonine kinase that plays a key role in mediating cell inflammation, autophagy, oxidative stress, and cell death [30,31]. Via the binding of the death domain on TNFRSF receptors followed by ligand stimulation, RIPK1 is recruited and forms an intracellular protein complex that could facilitate the activation of the canonical NF-κB pathway. As TNF-α is one of the most extensively studied inflammatory cytokines involved in neurodegenerative diseases. This review focuses on discussing TNF-α activated downstream pathways mediating NF-κB pathway activation and thereby demonstrates the key role of RIPK1 in this process. 

**Figure 1:**
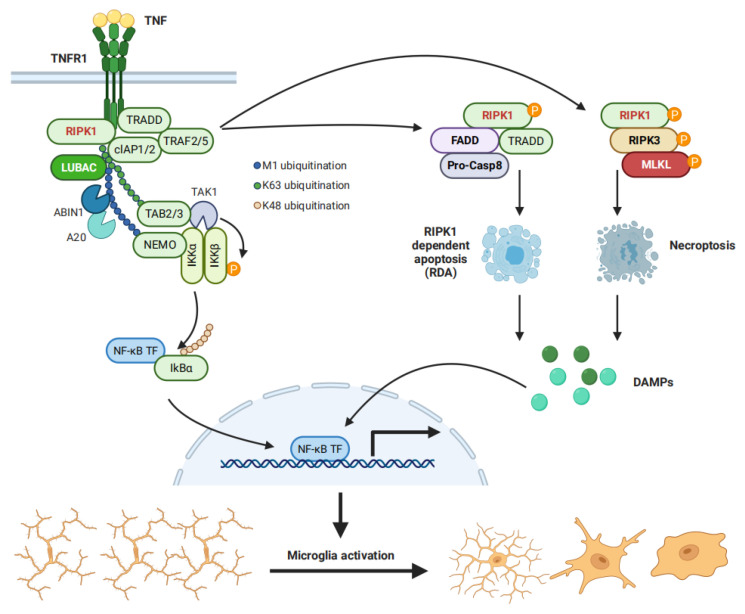
TNFR1 activation promotes NF-κB activation via cell-autonomous and non-autonomous mechanisms. TNF binds to TNFR1 to form an intracellular protein complex that promotes the activation of NF-κB via the scaffold function of RIPK1 (**Left**). The kinase activity of RIPK1 mediates RIPK1-dependent apoptosis (RDA) when cFLIPL expression induced by NF-κB is comprised, while activated RIPK1 promotes necroptosis when active caspases are inhibited (**Right**). Both RDA and necroptosis cause the release of damage-associated molecular patterns (DAMPs) to activate the NF-κB pathway via non-cell autonomous mechanisms.

The binding between TNF-α and tumor necrosis factor receptor 1 (TNFR1), a TNF receptor that is expressed prominently in multiple CNS cells, including microglia, leads to the recruitment of RIPK1 and TNFR1-associated death domain (DD) protein (TRADD) to the intracellular death domain of activated TNFR1 [32,33,34] and thus initiates the formation of complex I. Whereafter, TRADD recruits TNF receptor-associated factor 2 (TRAF2), which sequentially brings the E3 ubiquitin ligases cellular inhibitors of apoptosis 1 and 2 (cIAP1/2) into complex I. cIAP1/2 ubiquitinates RIPK1 in K63 and stimulates the recruitment of the linear ubiquitin assembly complex (LUBAC), adding M1-linked ubiquitin chains to RIPK1. The IKK complex then binds to the M1 ubiquitin chains on RIPK1. Meanwhile, K63 ubiquitin chains recruit transforming growth factor-β-activated kinase 1 (TAK1) to RIPK1 via a TAK1-binding protein 2 (TAB2) or TAB3. Eventually, these processes lead to the proximal interaction between TAK1 and IKKs, which allows TAK1 to phosphorylate IKKs and therefore degrades IκB and activates the NF-κB pathway [35,36] (Figure 1).

As the polyubiquitin chains of RIPK1 provide a scaffold for TAK1-mediated IKKs phosphorylation, it is reasonable to speculate that RIPK1 controls TNF-induced activation of the canonical NF-κB pathway. Indeed, RIPK1 deficiency in human skin fibroblasts, macrophages, and endothelial cells reduces NF-κB activation in response to TNF [37,38,39]. PARKIN, a major genetic cause of PD, mediates RIPK1 K63 ubiquitination on K376 and promotes TAK1 recruitment into complex I, thereby increasing the activation of NF-κB [35]. It is also notable that TNF induced NF-κB activation has been observed in RIPK1 knockout (KO) MEFs, although a high concentration of TNF used in this study might cause off-target effects that induce signal transductions through other receptors [40].

The scaffold function of RIPK1 in mediating NF-κB activation is now well-accepted; however, it remains unclear what the role of RIPK1 kinase activity on the NF-κB pathway is. RIPK1 kinase activity controls multiple cell death pathways, including apoptosis and necroptosis. Cell death can potentially release damage-associated molecular patterns (DAMPs) to activate both canonical and non-canonical NF-κB pathways on adjacent cells and propagate inflammation [23]. A recent study demonstrates that kinase-activated nuclear RIPK1 serves as a transcriptional coregulator that directly promotes the production of multiple inflammatory cytokines which may, in turn, mediate NF-κB pathway activation [41]. Therefore, RIPK1 kinase activity might contribute to NF-κB activation via non-cell-autonomous mechanisms (Figure 1)

## 4. RIPK1 Regulates Microglia Activity in Neurodegenerative Diseases

Given the fact that RIPK1 plays a critical role in modulating NF-κB activation via both cell-autonomous and non-autonomous mechanisms and that NF-κB pathways are indispensable for microglia to produce proinflammatory cytokines that generate inflammation and cause oxidative stress in neurodegenerative diseases [42,43], it is tempting to speculate that RIPK1 may be an important regulator of microglial neurotoxicity in promoting the pathological process of neurodegenerative diseases.

The expression and kinase activity of RIPK1 is indeed elevated in microglia in the context of neurodegenerative diseases [44,45,46]. AD and ALS patients exhibit elevated RIPK1 protein expression in CNS compared to age-matched controls, while mRNA expression levels of RIPK1 are unchanged [44,46]. Via double-staining with a microglial marker or culturing primary microglia derived from transgenic mice, increased RIPK1 protein expression in microglia has further been confirmed in neurodegenerative diseases. As the transcription of RIPK1 is comparable between the control and patient’s postmortem tissues, reasons that cause the up-regulation of RIPK1 protein might be due to impaired lysosomal or proteasomal activity involved in neurodegenerative pathogenesis [47,48,49]. Disturbed post-translational modifications or translation of mRNA in neurodegenerative diseases might also lead to the accumulation of RIPK1 protein [50,51]. Future studies are needed to explore the mechanisms involved in the elevated expression and subsequent activation of RIPK1 under neurodegenerative conditions.

**Figure 2:**
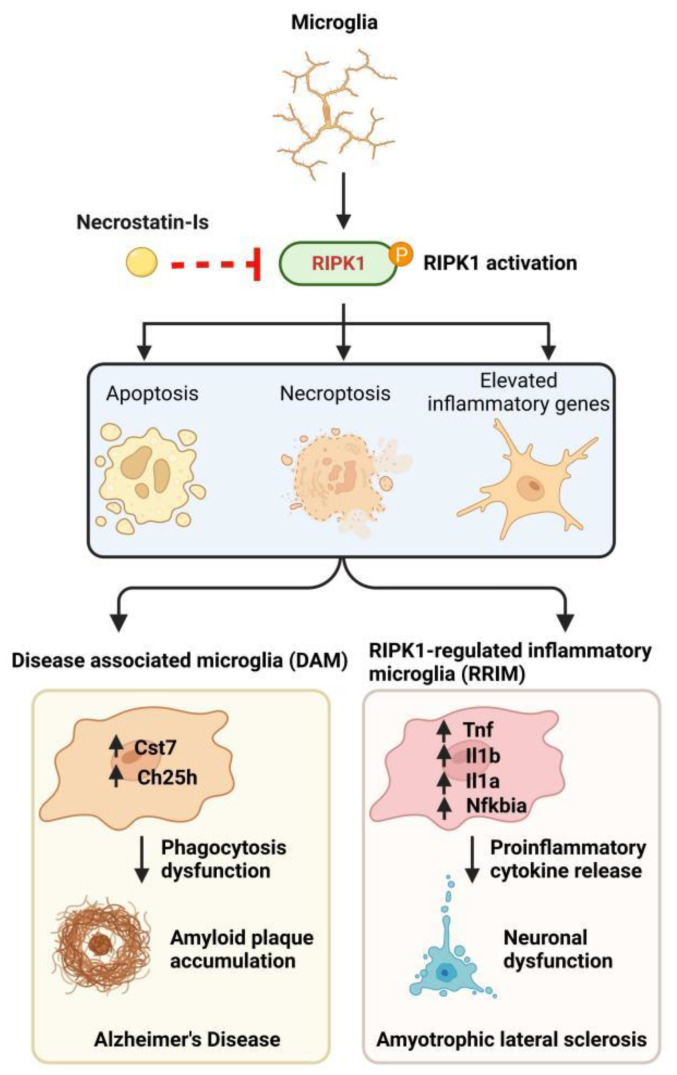
Activated RIPK1 promotes microglial apoptosis, necroptosis, and inflammatory response. In vitro, cell death-inducing agents stimulate RIPK1 kinase activity-dependent apoptosis, necroptosis, and inflammatory responses in microglia, all of which could be inhibited by the RIPK1 kinase activity inhibitor Nec-1s (**Upper**). In vivo, the inhibition of RIPK1 kinase activity ameliorates the DAM phenotype in AD and RRIM phenotype in ALS, respectively (**Lower**).

RIPK1-mediated microglial apoptosis or necroptosis has been demonstrated in vitro via the usage of well-established cell death-inducing agent TNF, plus 5Z-7-Oxozeaenol (O) or TNF stimulation with Smac mimetic and zVAD (Z), respectively, both of which could be effectively inhibited by the RIPK1 kinase activity inhibitor Nec-1s (Figure 2). Additional to microglial apoptosis and necroptosis, T/O and T/S/Z treatments also elevate the expression of inflammatory and NF-κB-regulated genes in microglia. This process is attenuated by Nec-1s, suggesting T/O or T/S/Z induces microglia-mediated inflammation in a kinase-dependent manner [52] (Figure 2).

In the brain of AD patients, there is an increased number of activated microglia clustered around the amyloid beta (Aβ) plaque [53], where they recognize and engulf Aβ which degraded in the microglial autophagic/lysosomal system later [54], through an ensemble of pattern recognition receptors (PRRs) on the microglia surface [55]. This phagocytosis process is, however, accompanied by the NF-κB activation-mediated expression and release of cell-toxic factors [56,57,58]. These cytokines not only contribute to neurotoxicity but also exhaust microglia and decreases their ability to clear Aβ [59]. A subtype of these exhausted microglia, named disease-associated microglia or degeneration-associated microglia (DAM), marked by the high expression of CH25H and CST7, have been associated with several neurodegenerative diseases, including AD [53]. *CST7* encodes a lysosomal inhibitor Cystatin F that may impair the microglial lysosomal pathway [60]. Highly expressed RIPK1 in microglia of human AD brains and an APP/PS1 mouse model is associated with induced CST7 expression, which may be contributed by increased RIPK1 kinase activity in AD, as the pharmacological or genetic inhibition of RIPK1 kinase activity in APP/PS1 mice reduces the DAM phenotype, as illustrated by the decrease of CST7 and restoration of microglial phagocytic activity [44,61]. In addition to the ability to regulate lysosomal function in microglia, activated RIPK1 also mediates cholesterol and lipid metabolism pathways. CH25H, a cholesterol hydroxylase, is markedly upregulated in DAM, which is principally responsible for the synthesis of 25-hydroxycholesterol (25HC), which may function as an inflammatory mediator to impact neural circuits and cognitive behaviors [62]. The expression of *CH25H* is attenuated by the inhibition of RIPK1, indicating that RIPK1 kinase activity interdicts some of the deleterious DAM phenotypes in AD (Figure 2).

Other than DAM, which is characterized by upregulated genes that impair lysosomal and phagocytic pathways while containing minor changes to a number of proinflammatory genes and NF-κB target genes, we recently have also identified a novel inflammatory microglia state in ALS that is regulated by RIPK1 kinase activity, named RIPK1-Regulated Inflammatory Microglia (RRIM) [63]. Unlike DAM, which highly expresses *Apoe*, *Trem2*, *Cd9*, *Cd63*, and *Cd52* genes, RRIM mainly expresses high levels of *Tnf*, *Il1a*, *Il1b*, *Ccl2*, *Ccl3*, *Ccl4*, *Tnfaip3*, and *Nfkbia*, the marker genes associated with inflammatory and NF-κB regulated classical proinflammatory pathways, which represent a highly proinflammatory microglia state (Figure 2). As discussed previously, increased RIPK1 kinase activity may induce inflammatory cytokine production via initiating cell death and release DAMPs or functioning as a proinflammatory gene transcriptional coregulator, or serving as a maker for active downstream signaling of TNFRSF receptors stimulated by multiple cytokines. Similar to DAM, the increased number of RRIM and the corresponding upregulated proinflammatory genes in mice with ALS are also decreased via genetic or pharmacological inhibition of RIPK1 kinase activity, suggesting RIPK1 kinase activity is a potential therapeutic target to ameliorate both DAM and RRIM-mediated neurotoxicity.

## 5. RIPK1 and ROS Activate Each Other in a Positive Feedback Loop

As discussed above, RIPK1 is activated in microglia, and the kinase activity of RIPK1 contributes to microglial dysfunction in multiple neurodegenerative diseases. Inhibiting RIPK1 kinase activity ameliorates the defects of microglial phagocytosis and the production of neurotoxic cytokines during neurodegeneration, while it remains largely unknown what causes or modulates the activation of RIPK1 in neurodegenerative diseases.

Elevated ROS in the brain is one of the most common hallmarks of various neurodegenerative diseases [64]. In AD, dysfunctional microglia decrease the ability to clear Aβ aggregates, which damage mitochondria and therefore increase ROS with elevated levels of lipid and protein peroxidation [65,66]. Additionally, antioxidants or antioxidant enzymes are decreased in AD patients, which may further lead to upregulated oxidative stress [67]. Patients with PD, which is characterized by neuronal loss in the substantia nigra (SN), exhibit reduced activity of mitochondrial Complex I in SN, which may facilitate the generation of ROS and induce nigra degeneration [68]. Biopsy samples from ALS patients also show mitochondrial defects and elevated mitochondrial ROS production that may lead to muscle pathology [69]. Collectively, these studies demonstrate that excessive ROS production may cause common pathophysiology in neurodegenerative diseases.

**Figure 3:**
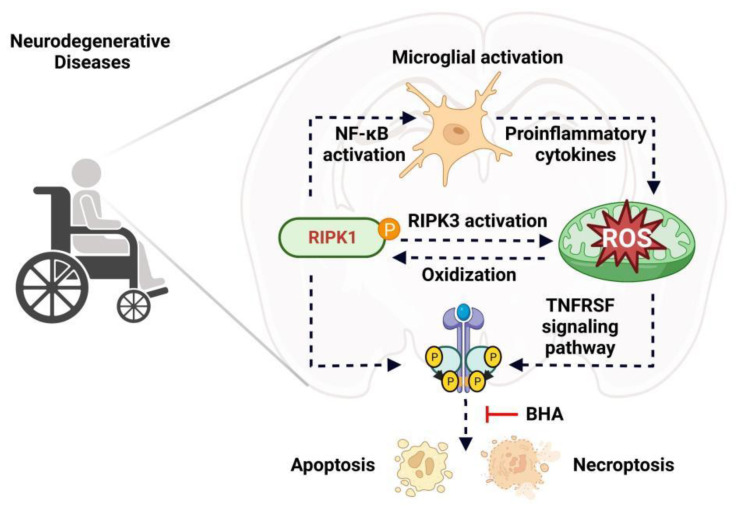
Activated RIPK1 and ROS generation enhance each other in a positive feedback loop. Excessive production of ROS activates RIPK downstream signaling pathways or directly oxidizes cysteine residues of RIPK1 to increase RIPK1 kinase activity. On the other hand, in neurodegenerative diseases, RIPK1 activation promotes ROS production via microglia-derived proinflammatory cytokines or activating RIPK3 that, in turn, phosphorylates the pyruvate dehydrogenase complex and increases ROS production.

ROS has been discovered to drive both necroptosis and apoptosis via regulating multiple signaling pathways mediated by TNFRSF receptors [70,71]. TNF-induced necroptosis could be efficiently blocked by butylated hydroxyanisole (BHA), a ROS scavenger [72]. In NF-κB activation deficient cells, BHA treatment effectively blocks RIPK1 kinase activity-dependent TNF-induced necroptosis without inhibiting the phosphorylation of RIPK1 [73]. Compression-induced oxidative stress sensitizes nucleus pulposus cells to apoptosis that could be blocked by either a RIPK1 inhibitor or an mPTP inhibitor, which has no impact on RIPK1 expression and activation [74]. In addition to acting downstream of RIPK1, a recent study has revealed that ROS could also directly oxidize three cysteine residues of RIPK1 that promote the autophosphorylation of RIPK1 on serine residue 161, a classical marker of RIPK1 kinase activity, and therefore enable the formation of RIPK1 and RIPK3 necrosome to initiate necroptosis [75] (Figure 3). All these findings suggest excessive ROS may cause microglial dysfunction via regulating RIPK1 and its downstream signaling in neurodegenerative diseases, which requires further detailed investigation in the near future. 

On one hand, excessive production of ROS activates RIPK1 and induces RIPK1-dependent necroptosis and apoptosis, while on the other hand, activated RIPK1 also promotes ROS generation under many pathological conditions of neurodegeneration. Inhibiting RIPK1 reduces ROS accumulation and microglial phenotypic alteration in cerebral ischaemic strokes [76]. Mechanistically, RIPK1 activation may increase ROS generation via regulating proinflammatory cytokine production. RIPK1 kinase activity-induced RRIM in ALS express high levels of *Tnf* and *Il1b* [63]. A TNF-α stimulation reduces mitochondrial membrane potential via recruiting Romo1 and Bcl-X_L_ to generate ROS, and IL-1β treatment increases ROS production depending on NADPH oxidase [77,78] (Figure 3). These data suggest RRIM-mediated TNF-α or IL-1β production might be the mechanistic link between RIPK1 activation and subsequent ROS generation.

Necroptosis, a major driver of neuronal cell death and microglial dysfunction in neurodegenerative diseases, is initiated by the subsequential TNF-α induced activation of RIPK1, RIPK3, and MLKL [79]. Activated RIPK3 involved in the necroptotic process phosphorylates pyruvate dehydrogenase complex to increase aerobic respiration and ROS production [80] (Figure 3). As inhibiting RIPK1 kinase activity totally abolishes TNF-α induced RIPK3 phosphorylation and activation, it is reasonable to speculate that activated RIPK1 might also facilitate the generation of ROS via activating RIPK3, which needs to be further investigated. Taken together, increased RIPK1 kinase activity promotes the generation of mitochondrial ROS that may compromise microglial homeostasis in neurodegenerative pathogenesis.

## 6. Conclusions

The up-regulation of RIPK1 expression and activity in neurodegenerative diseases has been demonstrated by numerous studies both in postmortem human samples and transgenic mice [44,46,52,63,81,82]. Particularly, inhibiting RIPK1 kinase activity ameliorates microglia-mediated neuroinflammation in AD, ALS, and MS, and prevents the development of phagocytosis defective microglia, DAM, inflammatory microglia, and RRIM. With growing evidence that RIPK1 plays a critical role in modulating microglia activity in neurodegeneration, there is an increasing number of RIPK1 inhibitors being investigated in human clinical studies for treating neurodegenerative diseases, such as ALS and AD [83].

To fully elucidate the critical role of RIPK1 kinase activity in regulating microglial homeostasis, it is ideal to target RIPK1 specifically in microglia. Via in vitro culturing murine microglia or human microglia from induced pluripotent stem cells (iPSC), RIPK1 kinase activity has been demonstrated to regulate microglial apoptosis, necroptosis, and proinflammatory signaling [52]. In vivo, specifically inhibiting RIPK1 in microglia is, however, still challenging. As deletion of RIPK1 leads to apoptotic death it is, therefore, infeasible to genetically inhibit microglial RIPK1 kinase activation via knocking out the RIPK1 gene. Pharmacologically inhibiting RIPK1, specifically in microglia, is now also not available. With the recent development of single-cell technologies, RIPK1-activated microglia in vivo might be identified and sorted to further investigate the mechanisms of RIPK1-mediated microglial dysfunction in the near future.

Unlike RIPK1-KO mice that die at birth with systemic inflammation, RIPK1 kinase inactive mice are viable, normally developed, and resistant to TNF-induced necroptosis [45,84]. These findings from transgenic mice suggest the clinical safety of inhibiting RIPK1 kinase activity. Indeed, many RIPK1 kinase inhibitors demonstrate safety profiles in clinical trials [83,85]. An ATP competitive RIPK1 kinase inhibitor GSK2982772 is now in clinical phase II for treating ulcerative colitis, and another RIPK1 inhibitor DNL758 has been initiated for phase II study in cutaneous lupus erythematosus patients. A blood–brain barrier permeable RIPK1 inhibitor DNL788 has been dosed in ALS patients in phase II clinical trial. Taken together, the RIPK1 inhibitor program now continues to be conducted for treating CNS and peripheral inflammation-related diseases with excellent clinical tolerance.

Although RIPK1 kinase activity and oxidative stress are closely regulated with each other in a vicious cycle, the molecular mechanisms that link RIPK1 activation and ROS generation are still not fully understood. NF-κB pathway activation, mediated by the scaffold function or kinase activity of RIPK1, promotes the release of multiple proinflammatory cytokines, which in turn damages mitochondrial homeostasis and enhances ROS generation. On the other hand, excessive production of ROS may also further activate RIPK1 and NF-κB via cell-autonomous and non-autonomous mechanisms that could play a detrimental role in neurodegenerative pathogenesis. Upregulated RIPK1 kinase activity and excessive ROS production may collude together, associating with NF-κB activation, to disturb microglial homeostasis in neurodegenerative diseases that still require further detailed studies.
